# Ergot and Digitalis in Hemorrhage

**Published:** 1856-12

**Authors:** 


*Ergot and Digitalis in Hemorrhage.*—A combination of ergot and digitalis, in the proportion of two grains of the former to three-quarters of a grain of the latter, given in the form of pill, six or eight times daily, is highly praised by M. Ed. Carriese, as an anti-hemorrhagic in general, and particularly in hæmoptysis and menorrhagia. According to him, this combination effects more than either article separately.—*Boston Med. & Surg. Journal.*

